# PLNFGL: joint estimation of multi-condition gene networks from single-cell RNA-seq data

**DOI:** 10.1093/bioinformatics/btag485

**Published:** 2026-07-03

**Authors:** Wenli Zhai, Dan Zhou, Zhongshang Yuan, Jiadong Ji

**Affiliations:** Institute for Financial Studies, Shandong University, Jinan, Shandong 250100, China; The Second Affiliated Hospital and School of Public Health, Zhejiang University School of Medicine, Hangzhou, Zhejiang 310058, China; The Second Affiliated Hospital and School of Public Health, Zhejiang University School of Medicine, Hangzhou, Zhejiang 310058, China; Department of Biostatistics, School of Public Health, Cheeloo College of Medicine, Shandong University, Jinan, Shandong 250100, China; Institute for Financial Studies, Shandong University, Jinan, Shandong 250100, China

## Abstract

**Motivation:**

Graphical models have been widely used in bioinformatics to infer the conditional dependence structure among random variables, but traditional Gaussian graphical models (GGMs) are suboptimal for single-cell RNA sequencing (scRNA-seq) due to dropout events and distributional mismatch. Moreover, most existing methods estimate networks under a single condition, limiting their utility in multi-condition studies.

**Results:**

We propose PLNFGL (Poisson Log-Normal Fused Graphical Lasso), a joint network estimation framework for scRNA-seq data. PLNFGL uses a multivariate Poisson log-normal model to accommodate dropout effects and estimates the covariance via moment methods. A joint graphical model is then employed to infer condition-specific precision matrices. Simulations show improved estimation accuracy. Applications to scRNA-seq data of Alzheimer’s disease and spatial transcriptomics of lung cancer reveal cell-type-specific interaction networks. Edge set enrichment enables pathway analysis, validating known interactions and highlighting novel disease-related targets. This work provides a powerful tool for the integrative analysis of scRNA-seq data.

**Availability and Implementation:**

The R implementation of PLNFGL is available at https://github.com/jijiadong/PLNFGL, and an archival version is available on Zenodo at https://doi.org/10.5281/zenodo.20744172.

## 1 Introduction

The probabilistic graphical model is a statistical framework that represents variable dependencies through graph structures and has been extensively applied in bioinformatics. Gaussian graphical models (GGMs) are a classical approach for modeling conditional independence in multivariate data. In GGMs, nodes represent variables and edges encode conditional dependencies. Under the assumption of joint normality, two variables are conditionally independent given all others if and only if the corresponding entry in the precision matrix (inverse covariance matrix) is zero (Lauritzen 1996). The network structure is therefore encoded in the sparsity pattern of the precision matrix. Numerous methods have been developed to estimate sparse precision matrices, including neighborhood selection with lasso regularization (Meinshausen and Bühlmann 2006), the *l*_1_-penalized log-likelihood maximization approach (Yuan and Lin 2007, Banerjee and El Ghaoui 2008, Friedman *et al*. 2008), and D-trace loss minimization (Zhang and Zou 2014). Covariate-adjusted GGMs have been proposed for learning directed acyclic graphs (DAGs) in genetical genomics, employing a two-stage estimation strategy that enables network inference while accounting for measured covariates (Gao *et al.* 2019). In addition, integrative graph-constrained modeling strategies have been developed to incorporate known network structures into statistical learning procedures, primarily to enhance phenotype-associated gene selection under structural constraints, rather than to focus explicitly on precision matrix estimation (Gao and Cui 2015).

In recent years, single-cell RNA sequencing (scRNA-seq) technology has revolutionized gene expression analysis by providing unprecedented resolution. However, the data complexity poses significant analytical challenges, primarily due to technical noise, low expression levels, and dropout events (Stegle *et al*. 2015, Zhang and Zhang 2020, Akers and Murali 2021). A major limitation of scRNA-seq is the high rate of missing values, which can represent “true zeros” (unexpressed genes) or “false zeros” resulting from sequencing failures. The latter type of zero caused by a sequencing failure is called a “dropout event” (Kharchenko *et al*. 2014, Hicks *et al.* 2018). Consequently, the Gaussian hypothesis is inappropriate for scRNA-seq count data and precision matrices inferred from GGMs may fail to accurately reflect actual variable interdependence. While alternative distributions, such as Poisson and negative binomial models (Allen and Liu 2013, Pan *et al.* 2023, Qiu *et al.* 2024, Chen *et al.* 2025), have been proposed to accommodate count data and over-dispersion, they often lack multivariate flexibility or robust theoretical proofs in hierarchical mixed models (Esnaola *et al.* 2013, Choudhary and Satija 2022). Although dropout events and zero-inflation are prominent challenges, recent literature suggests that after appropriate normalization, excess zeros can often be adequately explained by count models that properly account for overdispersion and latent correlation structures, rendering dedicated zero-inflation parameters unnecessary and potentially prone to identifiability issues (Townes *et al.* 2019, Silverman *et al.* 2020, Svensson 2020, Jiang *et al.* 2022). To address these modeling requirements, the multivariate Poisson log-normal (PLN) distribution offers a solution by assuming that observed counts follow a Poisson distribution conditioned on latent Gaussian variables (Aitchison and Ho 1989). This two-layer structure effectively captures gene-specific overdispersion and latent dependencies without assuming variable independence. By utilizing this hierarchical count-generating mechanism, the PLN framework implicitly accounts for many dropout-related zeros without requiring a separate zero-inflation process (Inouye *et al.* 2017, Silva *et al.* 2019).

Importantly, scRNA-seq data are often collected across multiple biologically related but distinct conditions, such as different cell types, disease states, or tissue contexts. Each condition may exhibit both shared and condition-specific gene interaction patterns due to conserved biological processes and context-dependent regulatory rewiring. Joint modeling across conditions can therefore improve statistical efficiency by borrowing strength, enhance robustness under sparse data, and facilitate the identification of both common and differential interaction structures (Danaher *et al*. 2014). Although several existing methods address network estimation under Gaussian assumptions or incorporate structural constraints, there remains a need for a unified framework that jointly infers gene interaction networks across conditions for count-based scRNA-seq data.

**Figure 1:**
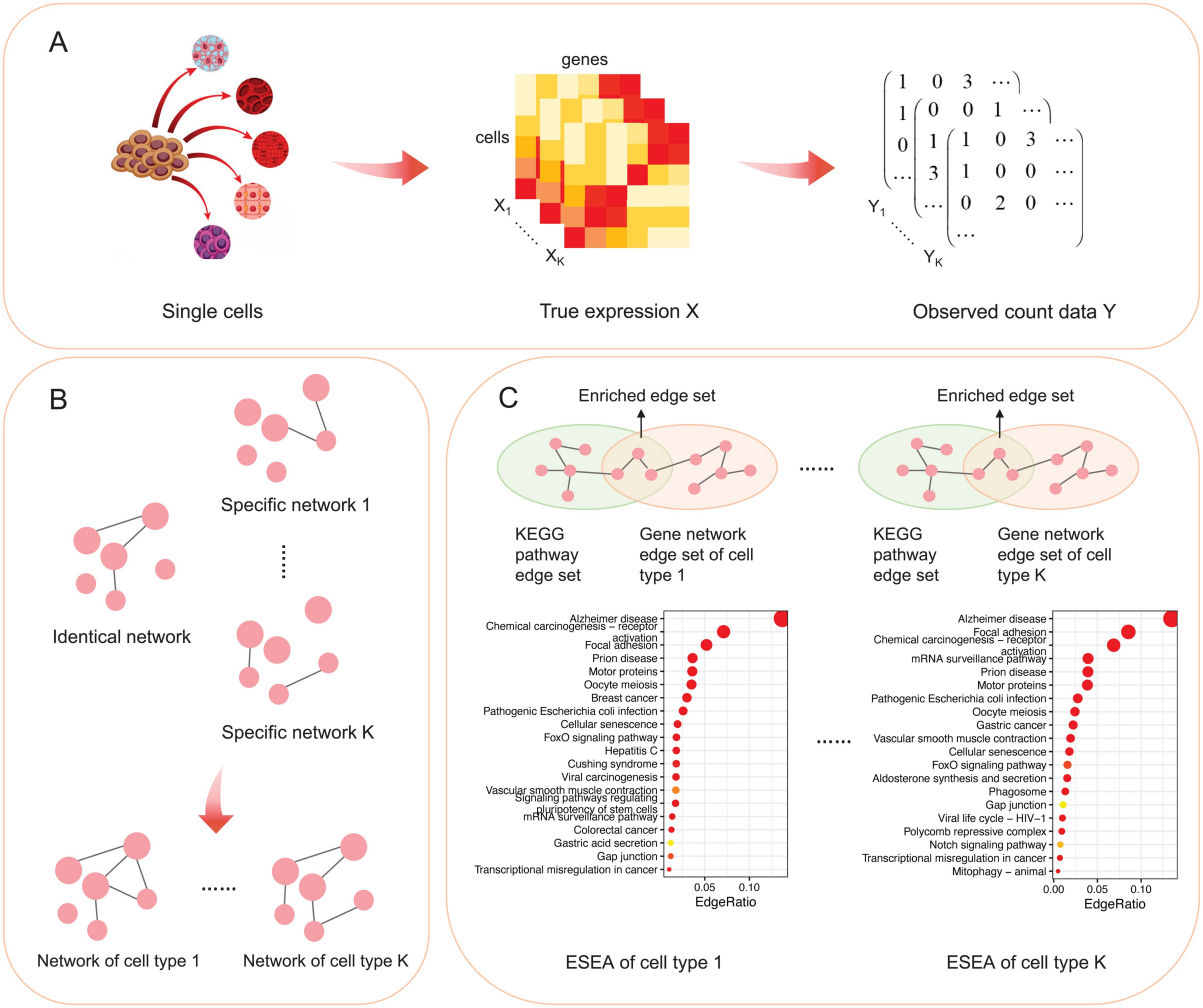
Illustration of PLNFGL framework. (A) Sampling of scRNA-seq data and estimation of the covariance matrix under dropout events. (B) Joint estimation of multiple gene interaction networks across conditions using the PLNFGL framework, which simultaneously captures shared and condition-specific network structures. (C) Illustration of the ESEA procedure for identifying significantly enriched pathways based on the inferred gene networks.

In this study, we propose PLNFGL (Poisson Log-Normal Fused Graphical Lasso), a framework for jointly estimating undirected gene interaction networks from scRNA-seq data. It uses a two-step approach: (i) modeling scRNA-seq data with the PLN distribution to account for technical noise and estimating covariance matrices via moment estimation (Fig. 1A); and (ii) inferring gene interaction networks under varying conditions, such as different cell types (Fig. 1B). By integrating shared information across conditions, the joint graphical model improves robustness and accuracy, yielding a comprehensive view of cell-type-specific interaction networks. To interpret these networks, we apply edge set enrichment analysis (ESEA) on the identified interaction edges (Fig. 1C), similar to gene set enrichment analysis (GSEA) (Subramanian *et al.* 2005). We validate PLNFGL on both simulated and real scRNA-seq datasets, demonstrating its superiority in recovering true interactions and its capability to identify cell-specific networks in Alzheimer’s disease (AD) and non-small cell lung cancer (NSCLC).

## 2 Materials and methods

### 2.1 The multivariate poisson log-normal graphical model

The distribution of scRNA-seq data is first modeled using the multivariate Poisson log-normal (PLN) graphical model, following the approaches of Aitchison and Ho (1989) and Xiao *et al.* (2022). Suppose ***Y***_*i*_ = (*Y_i1_*,…,*Y_ip_*)^*T*^ is the observed count vector of *p* genes in the *i*th sample cell, ***X***_*i*_ = (*X_i1_*,…,*X_ip_*)^*T*^ is the corresponding true expressions. Assume that conditional on ***X***_*i*_, *Y_ij_* (*j *= 1,…,*p*) are independent Poisson random variables with mean *S_i_X_ij_* (*j *= 1,…,*p*), where *S_i_* is a known scaling factor. In scRNA-seq data, *S_i_* represents the library size of the *i*th sample cell, which is related to the total sequencing reads and can be estimated by the sum of counts of each cell. The latent variable***X***_*i*_ follows a multivariate log-normal distribution with mean ***μ*** and covariance Σ. $\Theta =\Sigma^{-1}$ is the precision matrix, which represents the gene interaction network. Specifically, if $\Theta_{jl}\neq 0$, it means that there is an interaction relationship between the *j*th gene and *l*th gene. In summary, the PLN distribution can be represented in the following form:


(1) $$\begin{array}{c} Y_{i}|X_{i}∼\prod_{j=1}^{p}\mathrm{Poisson}\left(S_{i}X_{ij}\right),\\ \;\text{log}\;\left(X_{i}\right)∼N\left(\mu ,\Sigma \right). \end{array}$$

We denote this PLN distribution by $Y_{i}∼\text{PLN}\left(S_{i};\mu ,\Sigma \right)$.

### 2.2 Covariance moment estimator

(1) 's moments can be easily obtained through conditional expectation results and standard properties of the PLN distributions. Therefore, the moment estimation of Σ is $\Sigma^{*}={\left[\sigma_{jl}^{*}\right]}_{1\leq j,\;l\leq p}$, specifically,


(2) $$\sigma_{jl}^{*}=\left\{ \begin{array}{c} \;\text{log}\;\left(n^{-1}\sum_{i=1}^{n}\left[Y_{ij}\left(Y_{ij}-1\right)/{\left(S_{i}\right)}^{2}\right]\right)-2\;\text{log}\;\left({\hat{\alpha }}_{j}\right),\;\;1\leq j=l\leq p,\\ \;\text{log}\;\left(n^{-1}\sum_{i=1}^{n}\left[Y_{ij}Y_{il}/{\left(S_{i}\right)}^{2}\right]\right)-\text{log}\;\left({\hat{\alpha }}_{j}\right)-\text{log}\;\left({\hat{\alpha }}_{l}\right),1\leq j\neq l\leq p, \end{array}\right.$$

where ${\hat{\alpha }}_{j}=n^{-1}\sum_{i=1}^{n}Y_{ij}/S_{i}$ (Xiao *et al.* 2022), and *n* is the number of sample size.

To ensure positive definiteness, $\Sigma^{*}$ is projected into semi-positive definite matrix space, and a semi-positive definite matrix $\Sigma^{+}$ that is closest to $\Sigma^{*}$ is identified. Then a small positive definite matrix is added to $\Sigma^{+}$ to get a positive definite moment estimator $\hat{\Sigma }$ of Σ. specifically,


(3) $$\Sigma^{+}=\underset{A\geq 0}{\arg\min}{\left\|A-\Sigma^{*}\right\|}_{\infty },$$

(4) $$\hat{\Sigma }=\Sigma^{+}+{\left\|\Sigma^{+}-\Sigma^{*}\right\|}_{\infty }I,$$

where *A *≥ 0 means *A* is a semi-positive definite matrix, ${\left\|A\right\|}_{\infty }=\underset{i,j}{\max}\left|A_{ij}\right|$, *I* is a *p *×* p* identity matrix. The optimization problem (3) for $\Sigma^{+}$ can be solved by a splitting conic solver (Fu *et al*. 2020).

### 2.3 Joint Poisson log-normal graphical model

Suppose $Y^{\left(1\right)},\ldots ,Y^{\left(K\right)}$ are the observed *K* (*K *≥ 2) sequencing datasets, where ***Y***^(^^*k*^^)^ (*k *= 1,…,*K*) is an *n_k_*×*p* count matrix consisting of *n_k_* observations with measurements on a set of *p* genes, $Y_{i}^{\left(k\right)}∼\text{PLN}\left(S_{i}^{\left(k\right)};\mu^{\left(k\right)},\Sigma^{\left(k\right)}\right)$. Firstly, ${\hat{\Sigma }}^{\left(k\right)}\left(k=1,\ldots ,K\right)$ are estimated by moment estimator in 2.2 section. Then the fused graphical lasso (FGL) (Danaher *et al*. 2014) is employed to estimate $\Theta^{\left(k\right)}={\left(\Sigma^{\left(k\right)}\right)}^{-1}\left(k=1,\ldots ,K\right)$ by embedding ${\hat{\Sigma }}^{\left(k\right)}\left(k=1,\ldots ,K\right)$ into FGL, which has a penalized log-likelihood form:


(5) $${\text{maximize}}_{\left\{\Theta \right\}}\left(\sum_{k=1}^{K}n_{k}\left[\text{log}\;\left\{\det\left(\Theta^{\left(k\right)}\right)\right\}-\text{tr}\left({\hat{\Sigma }}^{\left(k\right)}\Theta^{\left(k\right)}\right)\right]-P\left(\left\{\Theta \right\}\right)\right),$$

where tr(*A*) is the trace of matrix *A*, $\Theta =\left(\Theta^{\left(1\right)},\ldots ,\Theta^{\left(K\right)}\right)$, $\Theta^{\left(k\right)}={\left[\theta_{ij}^{\left(k\right)}\right]}_{1\leq i,j\leq p}$. P({Θ}) is a convex penalty function, which has the following form:


(6) $$P\left(\left\{\Theta \right\}\right)=\lambda_{1}\sum_{k=1}^{K}\sum_{i\neq j}\left|\theta_{ij}^{\left(k\right)}\right|+\lambda_{2}\sum_{k<k^{\prime }}\sum_{i,j}\left|\theta_{ij}^{\left(k\right)}-\theta_{ij}^{\left(k^{\prime }\right)}\right|,$$

where *λ*_1_ and *λ*_2_ are tuning parameters. *λ*_1_ controls the sparsity of the estimated precision matrix ${\hat{\Theta }}^{\left(1\right)},\ldots ,{\hat{\Theta }}^{\left(K\right)}$. The larger *λ*_1_ is, the sparser ${\hat{\Theta }}^{\left(1\right)},\ldots ,{\hat{\Theta }}^{\left(K\right)}$ will be. *λ*_2_ controls the similarity between ${\hat{\Theta }}^{\left(1\right)},\ldots ,{\hat{\Theta }}^{\left(K\right)}$. Many elements of ${\hat{\Theta }}^{\left(1\right)},\ldots ,{\hat{\Theta }}^{\left(K\right)}$ will be identical across groups when *λ*_2_ is large. That is, FGL borrows information across groups to encourage not only similar network structures but also similar edge values.

For tuning parameter selection, in simulation study, a range of *λ*_1_ and *λ*_2_ are selected to study the performance of PLNFGL. In general, *λ*_1_ and *λ*_2_ are controlled at (0, 1.5]. In real data analysis, Akaike information criterion (AIC) is used to select tuning parameters for PLNFGL, it has the following form:


(7) $$\text{AIC}\left(\lambda_{1},\lambda_{2}\right)=\sum_{k=1}^{K}\left[n_{k}\text{tr}\left({\hat{\Sigma }}^{\left(k\right)}{\hat{\Theta }}_{\lambda_{1},\lambda_{2}}^{\left(k\right)}\right)-n_{k}\;\text{log}\;\left\{\det\left({\hat{\Theta }}_{\lambda_{1},\lambda_{2}}^{\left(k\right)}\right)\right\}+2E_{k}\right],$$

where ${\hat{\Theta }}_{\lambda_{1},\lambda_{2}}^{\left(k\right)}$ is the precision matrix of the *k*th group estimated using *λ*_1_ and *λ*_2_, *E_k_* is the number of nonzero elements in ${\hat{\Theta }}_{\lambda_{1},\lambda_{2}}^{\left(k\right)}$.

### 2.4 Edge set enrichment analysis

To explore the biological themes underlying cell-type-specific interactions, we performed an edge set enrichment analysis inspired by clusterProfiler (Yu *et al.* 2012) and ESEA (Han *et al.* 2015). A total of 354 pathways’ information in the KEGG PATHWAY database (Release 118.0) is downloaded from https://www.genome.jp/kegg. Each pathway is converted to an undirected graph, where genes contained in the pathway serve as nodes and are connected in pairs as edges. These edges form a background set for enrichment. This fully connected design intentionally facilitates the discovery of novel gene-gene interactions beyond curated knowledge. To ensure that this densified background does not artifactually influence our findings, we validated key results using a strictly curated, biologically annotated interaction network as the background edge set (see S1 section in the Supplementary Data). PLNFGL is then applied to jointly infer the gene interaction networks across different cell types. For each cell type, to determine whether any term annotates the specified edge list with a higher frequency than expected by chance, we calculate the *P* value using the hypergeometric distribution:


(8) $$p-\mathrm{value}=1-\sum_{k=0}^{k_{ij}-1}\frac{\left(\begin{array}{c} M_{i}\\ k \end{array}\right)\left(\begin{array}{c} N-M_{i}\\ n_{j}-k \end{array}\right)}{\left(\begin{array}{c} N\\ n_{j} \end{array}\right)},$$

where *N* is the total number of edges in the background edge set, *n_j_* is the total number of identified edges in all terms of the *j*th cell type, *M_i_* is the total number of edges in the *i*th term, *k_ij_* is the number of identified edges in the *i*th term of the *j*th cell type. *P* value is multiple adjusted. Only terms with a Benjamin-Hochberg adjusted *p*-value < 0.05 are considered significant.

For illustration, suppose that the background edge set contains *N *= 10,000 edges. In a given cell type *j*, suppose PLNFGL identifies *n_j_* = 500 edges in total. For a specific pathway *i*, assume that this pathway contains *M_i_* = 200 edges in the background network, and that *k_ij_* = 25 of the 500 identified edges fall within this pathway. Equation (8) then computes the probability of observing at least 25 overlapping edges between the identified edge set and the pathway purely by chance. A small resulting *P-*value indicates that the pathway is significantly enriched in the inferred network for that cell type.

### 2.5 Alzheimer’s disease (AD) and non-small cell lung cancer (NSCLC) datasets

To evaluate our proposed methodology, we utilized two distinct, publicly available transcriptomic datasets: a scRNA-seq dataset of AD (Grubman *et al.* 2019) and a spatial molecular imaging (SMI) dataset of NSCLC (He *et al.* 2022). Because these datasets were obtained from thoroughly validated, previously published studies, we directly downloaded and utilized their final, quality-controlled, and normalized expression matrices for our downstream analysis. Briefly, the AD dataset was originally derived from human post-mortem entorhinal cortex tissues. The original preprocessing pipeline included doublet removal, strict filtering of cells based on mitochondrial fraction (<10%) and library size (excluding the top and bottom 5% of UMI counts), and global scaling normalization. The NSCLC spatial dataset was generated using a high-plex imaging platform. The original data underwent strict background noise filtering, spatial deduplication, and removal of segmented cells containing fewer than 20 total transcripts before library-size normalization.

## 3 Results

### 3.1 Simulation settings

We evaluated PLNFGL against five competing methods: PLNet (Xiao *et al.* 2022), JGNsc (Dong *et al.* 2023), glasso (Friedman *et al*. 2008), FGL (Danaher *et al*. 2014), GGL (Danaher *et al*. 2014). We assess these methods under various conditions, including different dropout rates, sample sizes, dimensions, numbers of groups. The library sizes are generated from a log-normal distribution with mean log10 and standard deviation 0.1, and are estimated by the sum of counts of each cell. Three graph structures are considered:

1. Scale-free graph: Generated using the Barabási—Albert model (Barabási and Albert 1999) with a power parameter 1.
2. Random graph: Constructed by randomly connecting pairs of nodes with a probability of 1/*p*.
3. K-nearest-neighbor (KNN) graph: Created by modifying the data generation mechanism described by Guo *et al*. (2011), with *k* = 3 in our simulation.

Each graph corresponds to a *p*×*p* precision matrix. The nonzero element values are drawn from a uniform distribution with support on [0.2, 0.4]. The diagonal elements are set as 1. If the precision matrix is not positive definite, a constant of 0.1 is added to its diagonal to ensure positive definiteness. In our benchmark setting, we set *n *= 200, *p *= 100, dropout rate (the ratio of zero in count matrix) of 30%, *K *= 3. Three-group networks are generated, where scale-free and random networks adopt block-diagonal structures of 10 equally sized blocks, while KNN networks are divided into 10 virtual blocks for consistency. Of these, eight blocks are shared across groups, one is shared by two groups, and one is unique to a single group.

We use precision-recall (PR) curves to evaluate the performance of various simulations. Precision and recall are defined as follows:


(9) $$\begin{array}{c} \text{precision}=\frac{\sum_{k=1}^{K}\sum_{i<j}Ι\left(\left|\theta_{ij}^{\left(k\right)}\right|\neq 0\right)Ι\left(\left|{\hat{\theta }}_{ij}^{\left(k\right)}\right|>\varepsilon \right)}{\sum_{k=1}^{K}\sum_{i<j}Ι\left(\left|{\hat{\theta }}_{ij}^{\left(k\right)}\right|>\varepsilon \right)},\\ \text{recall}=\frac{\sum_{k=1}^{K}\sum_{i<j}Ι\left(\left|\theta_{ij}^{\left(k\right)}\right|\neq 0\right)Ι\left(\left|{\hat{\theta }}_{ij}^{\left(k\right)}\right|>\varepsilon \right)}{\sum_{k=1}^{K}\sum_{i<j}Ι\left(\left|\theta_{ij}^{\left(k\right)}\right|\neq 0\right)}. \end{array}$$

where I(·) is an indicator function, $\theta_{ij}^{\left(k\right)}$ is the (*i*, *j*)-th entry of $\Theta^{\left(k\right)}\left(k=1,\ldots ,K\right)$, ${\hat{\theta }}_{ij}^{\left(k\right)}$ is the (*i*, *j*)-th entry of ${\hat{\Theta }}^{\left(k\right)}\left(k=1,\ldots ,K\right)$. In the benchmark setting, we set *ε *= 10^−3^ to define the presence of an estimated edge.

### 3.2 PLNFGL outperforms competing methods in simulations

**Figure 2:**
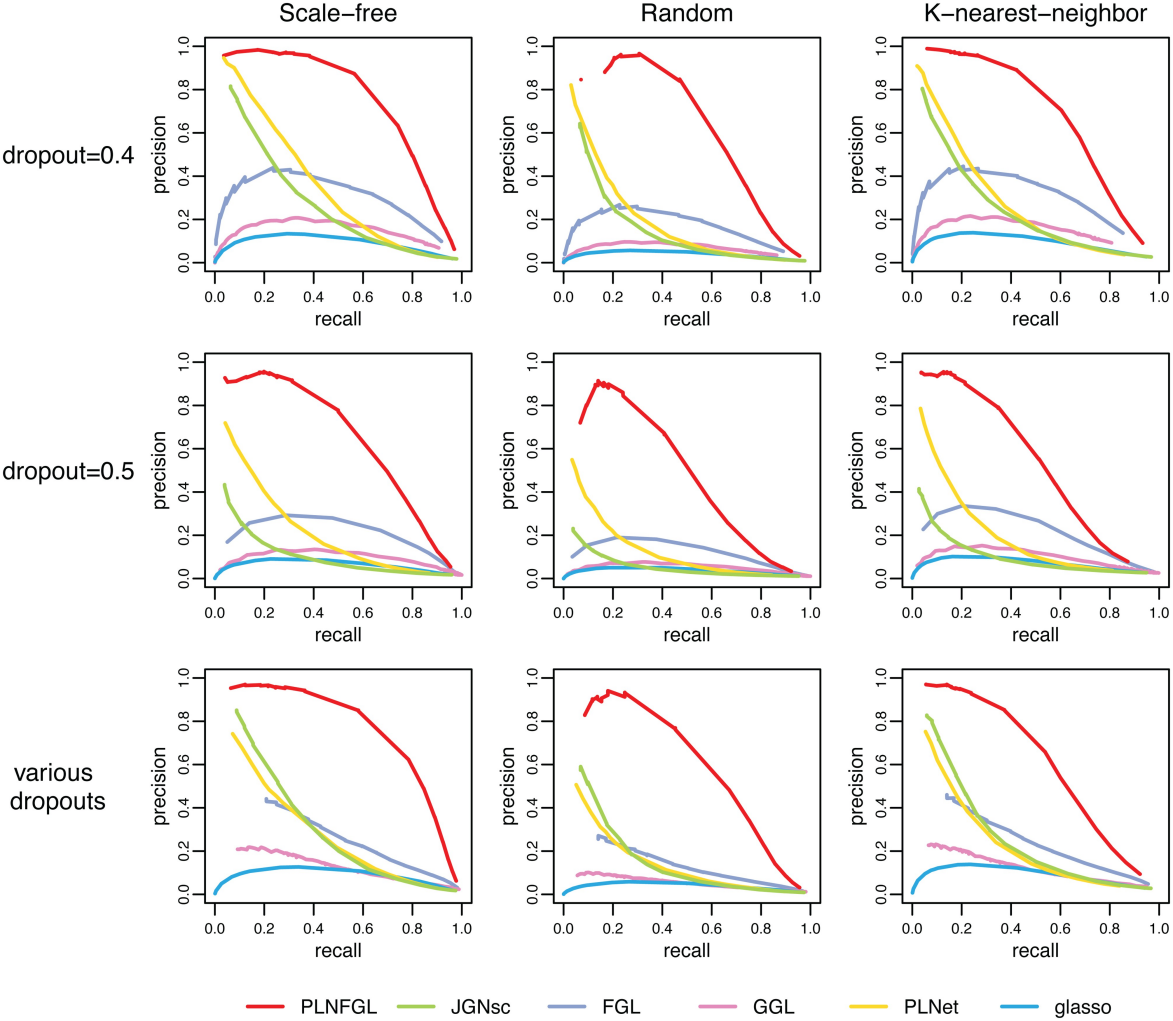
Simulation performance of different methods under varying dropout rates. Simulation settings were fixed at *n *= 200, *p *= 100, and *K *= 3, with dropout rates of 0.4, 0.5, and {0.3, 0.4, 0.5}. Rows correspond to different dropout scenarios, while columns represent different underlying graph structures.

First, we evaluate six network inference methods under three dropout rate scenarios: a fixed 40% dropout rate (***μ*** = −2.36 for all cells), a fixed 50% dropout rate (***μ*** = −2.76 for all cells), and a heterogeneous setting in which each cell is randomly assigned a dropout rate of 30%, 40%, or 50% with equal probability (corresponding to ***μ*** ∈ {−1.95, −2.36, −2.76}). Figure 2 shows PR curves under different dropout rate conditions, with results averaged over 50 simulation replicates. As shown in Fig. 2, PLNFGL consistently outperforms other methods across all dropout rates and graph structures, particularly under lower dropout. Among scRNA-seq-oriented approaches, PLNFGL, PLNet, and JGNsc surpass GGMs, with PLN- based models (PLNFGL and PLNet) outperforming the Bayesian zero-inflated Poisson model of JGNsc. JGNsc performance declines more under higher dropout compared to PLNFGL and PLNet. Among GGMs, the joint estimation methods of JGL outperform glasso, which estimates each network independently. In summary, joint estimation methods outperforms method for estimating each network individually, and the PLN graphical model fits scRNA-seq data better than GGM and Bayesian zero-inflated Poisson model.

**Figure 3:**
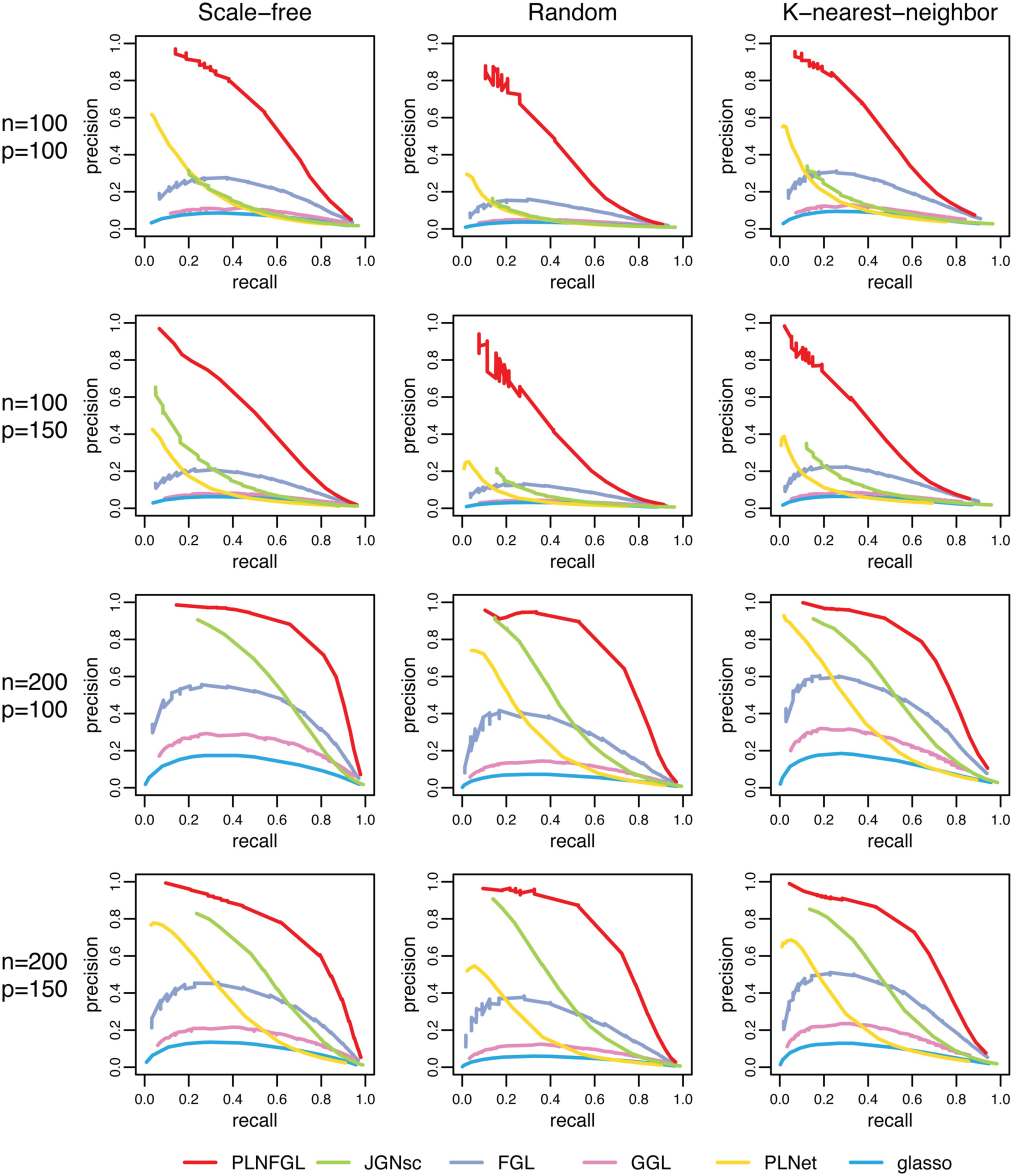
Simulation performance under different sample sizes and feature dimensions. Simulation settings included (*n*, *p*) = (100, 100), (100, 150), (200, 100), (200, 150), with *K *= 3 and a dropout rate of 0.3. Rows correspond to different values of (*n*, *p*), while columns correspond to different graph structure.

Next, we evaluate the six methods under varying sample sizes and dimensionalities. We consider relatively small sample sizes (*n *= 100, 200) with *p *>* n*. Figure 3 shows PR curves for *n* = {100, 200} and *p* = {100, 150}. PLNFGL performs well when *n *>* p* and maintains reasonable results in high-dimensional settings. As expected, lower sample size and higher dimensionality reduce precision and recall due to increased noise and redundancy. Additionally, the fluctuations observed on the left end of the PLNFGL PR curves when *p *>* n* are reasonable and anticipated. Under such high-dimensional settings, the large tuning parameter *λ*_1_ enforces high sparsity in the precision matrix, making the precision and recall ratios highly sensitive to the addition or removal of even a single edge.

**Figure 4:**
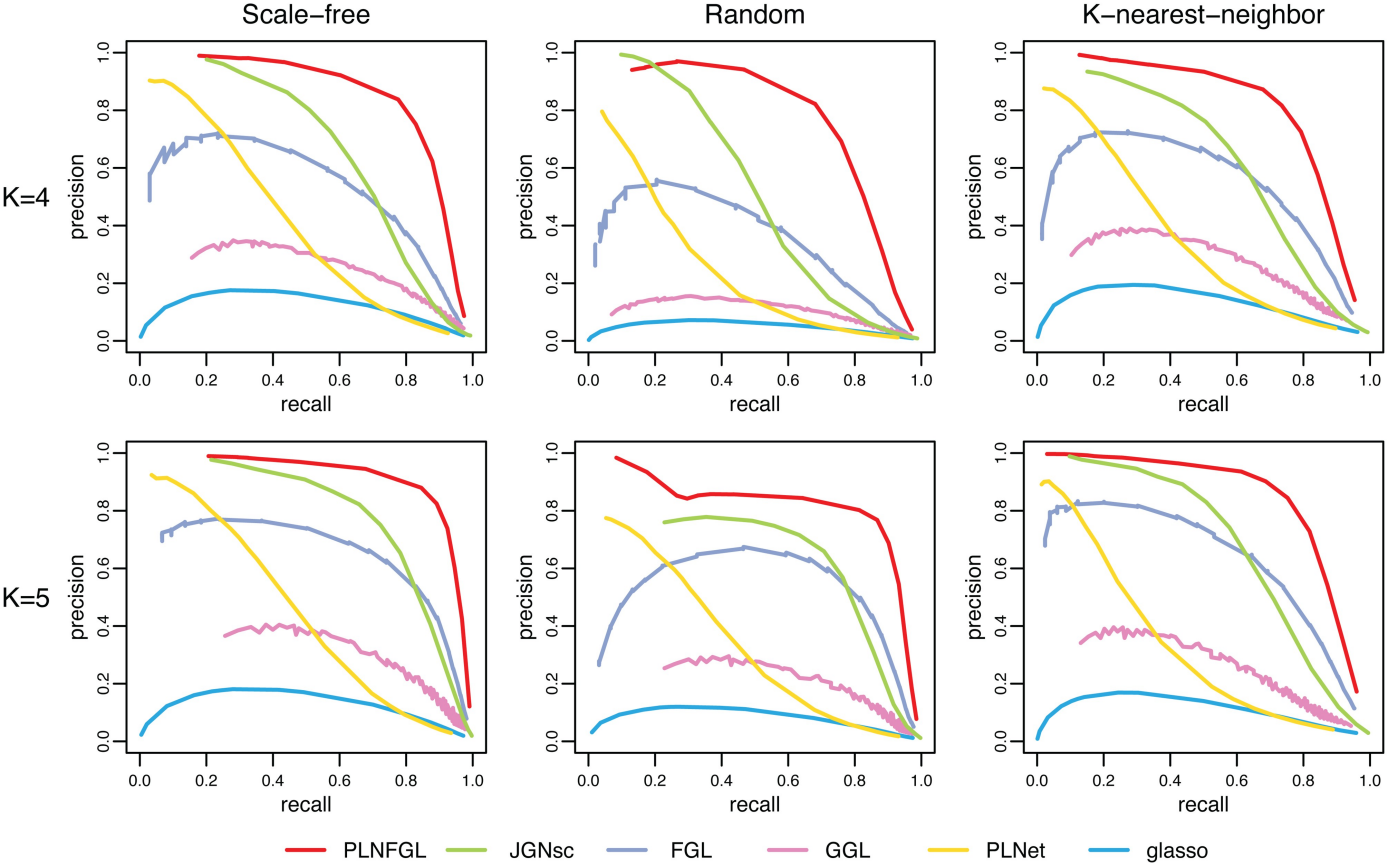
Simulation performance under different numbers of groups. Simulation settings were fixed at *n *= 200, *p *= 100, and dropout rate = 0.3, while the number of groups was varied (*K *= 4 and *K *= 5). Rows correspond to different numbers of groups, while columns correspond to different graph structure.

To evaluate joint estimation across multiple networks, we examined group numbers *K *= 4 and 5, where the fourth precision matrix equals the first, and the fifth equals the second. As shown in Fig. 4, increasing *K* improves performance. PLNFGL shows better PR curves for *K *= 4 or 5 than for *K *= 3. Joint estimation methods (PLNFGL, JGNsc, FGL, GGL) benefit from more groups, while PLNet and glasso, which estimate networks separately, show no clear improvement. This suggests joint estimation is superior when handling multiple groups.

Furthermore, we evaluated method robustness against variations in library sizes (see S2 section in the Supplementary Data) and edge selection thresholds (i.e. *ε*. see S3 section in the Supplementary Data). As shown in Fig. S3, scRNA-seq-specific methods (PLNFGL, PLNet, and JGNsc) exhibit strong robustness to library size variations, whereas Gaussian-based approaches suffer significant performance degradation as variation increases. Additionally, sensitivity analyses (Fig. S4) indicate that varying the selection threshold has minimal impact on precision and recall. Crucially, the relative performance rankings among the methods remain entirely consistent across all tested cutoffs, preserving the comparative advantages of PLNFGL. Overall, PLNFGL consistently outperforms competing methods across diverse parameter settings, confirming its superior accuracy and robust stability under varying analytical conditions.

### 3.3 Real data analyses on Alzheimer’s disease and non-small cell lung cancer

AD is a complex multifactorial heterogeneous pathology. Single-cell analysis has revealed that Alzheimer’s dementia involves intricate interactions among all major brain cell types (Murdock and Tsai 2023). Here we apply PLNFGL to an scRNA-seq dataset (GEO accession number GSE138852) of AD (Grubman *et al.* 2019) with 13,214 single nuclei from six AD and six control brains, focusing on 5,727 AD cells classified into five major types—4,655 oligodendrocytes (oligo), 472 astrocytes (astro), 249 neurons (neuron), 179 oligodendrocyte progenitor cells (OPC), and 172 microglia (mg). Only pathways with at least 10 shared genes are included, resulting in a total of 255 pathways. Then PLNFGL is applied to jointly infer the gene interaction networks across five cell types in each term, with tuning parameters selected using AIC criterion of Equation (7). Besides, the other five comparison methods in simulation studies perform the same analysis.

**Figure 5:**
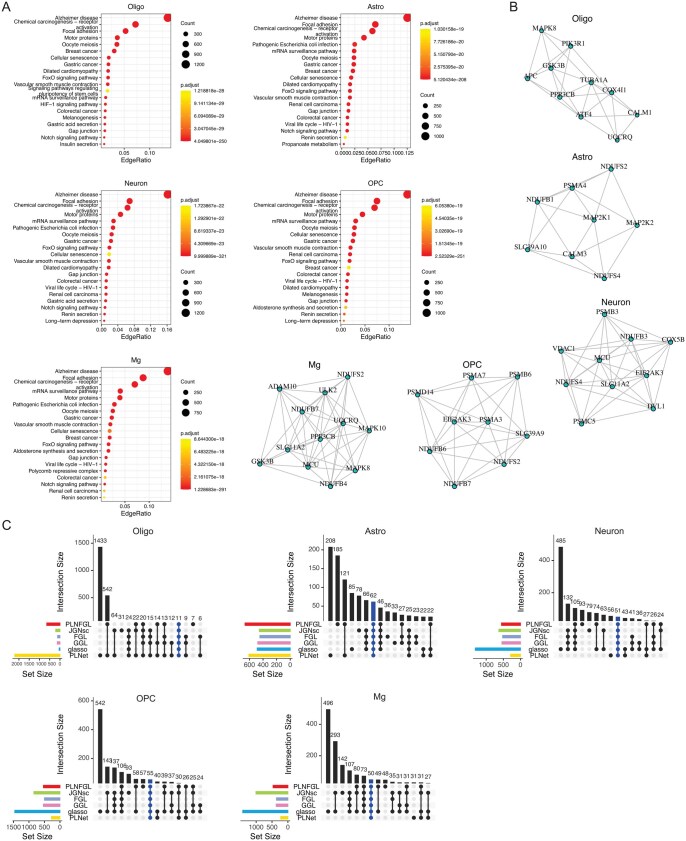
Analysis of Alzheimer’s disease (AD) scRNA-seq data. (A) Top 20 significantly enriched pathways identified by ESEA for each cell type based on PLNFGL-inferred gene interaction networks. (B) Gene interaction network of AD pathway for each cell type. (C) Upset plots showing the overlap of identified iGenes across six network inference methods for each cell type. Vertical bars represent the number of overlapping or unique iGenes, with the blue vertical bar specifically highlighting the iGenes commonly identified by all six methods. Connected dots indicate the corresponding combinations of methods, while the horizontal bars denote the total number of iGenes identified in the right matrix by each respective method.

First, we apply ESEA to identify the KEGG pathways related to AD. With *p*-adjust < 0.05 significance threshold, ESEA yield 39 pathways with statistical significance in oligo, 45 in astro, 43 in neuron, 43 in OPC, and 40 in mg (Table S1). The AD pathway is consistently significant, as are focal adhesion and chemical carcinogenesis receptor activation pathways (Fig. 5A), aligning with prior findings (Caltagarone *et al*. 2007, Lukiw and Pogue 2007, Monte de la *et al.* 2009). Certain pathways show cell-type enrichment, for example, N-glycan biosynthesis in microglia and SNARE interactions in vesicular transport in oligodendrocytes. Extensive evidence has demonstrated that aberrant glycosylation disrupts immune homeostasis, activates microglia, and promotes the release of inflammatory mediators, thereby exacerbating neuroinflammatory responses (Cheng *et al*. 2025). Oligodendrocytes are responsible for forming and maintaining the myelin sheaths that envelop neuronal axons, and they can utilize extracellular vesicles as mediators of intercellular communication (Reiter and Bongarzone 2020). Our results further suggest that oligodendrocytes may be involved in SNARE interactions. Across cell-type-specific AD pathway interaction networks, we observed extensive interactions involving the well-established AD risk-associated NDUF gene family (Rimal *et al.* 2024) (Fig. 5B). Additionally, we uncovered a novel three-way interaction in microglia involving *GSK3B*, *MCU*, and *NDUFB4*. These genes converge on interconnected aspects of mitochondrial biology—metabolic regulation (*GSK3B*), calcium handling (*MCU*), and respiratory chain function (*NDUFB4*). To our knowledge, this tripartite interaction has not been previously reported, suggesting a previously unrecognized regulatory node that may coordinate metabolic signaling, calcium dynamics, and oxidative phosphorylation in microglia, with potential relevance to Alzheimer’s pathogenesis. We define iGenes that connect more than 5 genes in a network. The upset plots revealed substantial overlap of iGenes identified across methods, with an average of 42.7% of the iGenes detected by PLNFGL being replicated by at least one other method (Fig. 5C). The greatest overlap was observed in astrocytes, whereas oligodendrocytes showed comparatively lower agreement among methods.

**Figure 6:**
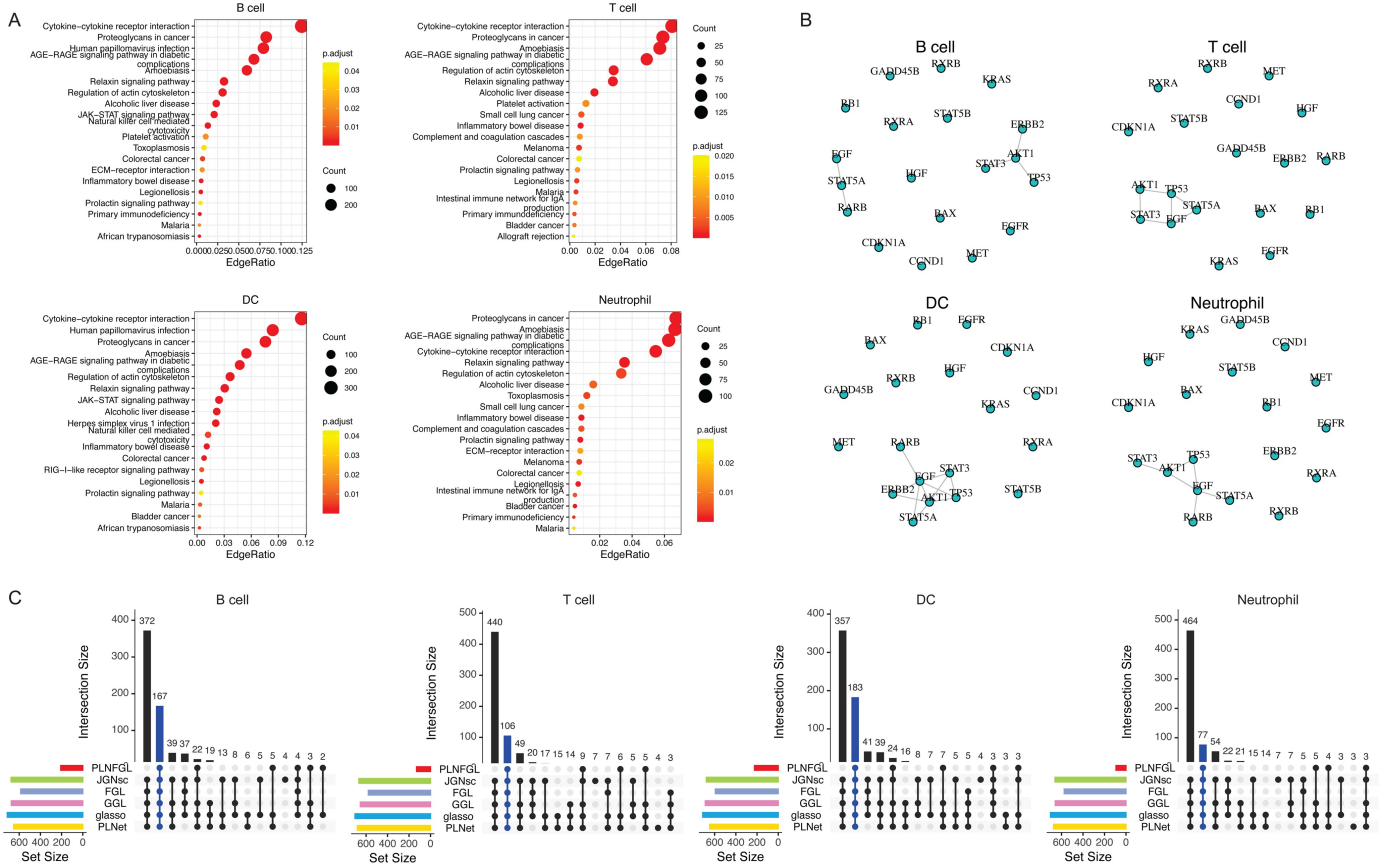
Analysis of non-small cell lung cancer (NSCLC) spatial transcriptomic data. (A) Top 20 significantly enriched pathways identified by ESEA for each cell type based on PLNFGL-inferred interaction networks. (B) Gene interaction network of NSCLC pathway for each cell type. (C) Upset plots showing the overlap of identified iGenes across six network inference methods for each cell type. Vertical bars represent the number of overlapping or unique iGenes, with the blue vertical bar specifically highlighting the iGenes commonly identified by all six methods. Connected dots indicate the corresponding combinations of methods, while the horizontal bars denote the total number of iGenes identified in the right matrix by each respective method.

Next, we apply PLNFGL to spatial transcriptomics data from NSCLC. The dataset, from one of eight FFPE NSCLC tissue samples on the NanoString website (http://nanostring.com/CosMx-dataset) (He *et al.* 2022), includes 980 genes measured across 91,972 single cells with cell type. We focus on immune cells in tumors, extracting 3,953 B cells, 7,857 T cells, 2,069 dendritic cells (DCs), and 5,929 neutrophils. The analysis follows the same approach as the AD study. A total of 166 pathways are analysed. With *p*-adjust < 0.05 pathway significance threshold, ESEA yield 20 pathways with statistical significance in B cells, 22 in T cells, 19 in DCs, and 22 in neutrophils (Table S2). Figure 6A shows the first 20 enriched pathways of each cell type. Among these, cytokine-cytokine receptor interaction and proteoglycans in cancer are consistently significant, consistent with previous NSCLC reports (Dong *et al.* 2020, Wang *et al.* 2020, Guo *et al.* 2022). Certain pathways exhibit cell-type specificity, including basal cell carcinoma pathway in T cells, and RIG-I-like receptor signaling pathway in dendritic cells. In the gene interaction networks of NSCLC pathway (Fig. 6B), PLNFGL identifies conserved hub genes (e.g. *TP53*, *AKT1*, *STAT3*, *STAT5A*, *EGF*) while revealing cell-type-specific network rewiring. Notably, a *STAT5A*-*TP53* interaction is uniquely detected in T cells, consistent with prior evidence in Treg regulation (Cui *et al.* 2023), whereas a distinct *STAT5A*-*RARB* interaction is specific to B cells, representing a previously uncharacterized regulatory relationship. The upset plots of iGenes across all pathways and cell types demonstrated considerable overlap among the six methods, suggesting that major regulatory hub genes were consistently detected (Fig. 6C). Compared with competing approaches, PLNFGL identified an average of 63.1% of iGenes per cell type that were replicated by at least one other method, compared to only 12.8%–15.7% for the alternative methods.

## 4 Discussion

This study introduces a joint estimation model for gene interaction networks based on scRNA-seq data. Compared to GGMs, the PLN distribution is more suitable for scRNA-seq data, particularly in addressing the dropout events. We propose PLNFGL, a two-step method for estimating precision matrices across multiple groups. The covariance matrix is first estimated using moment estimation, and then the precision matrix is jointly estimated by minimizing the penalized log-likelihood. A significant advantage of PLNFGL is that it does not require additional computational developments. Specifically, existing algorithms can be directly applied to estimate the positive definite covariance matrix Σ. Xiao *et al.* have proved that $\hat{\Sigma }$ is a consistent estimator for Σ. Secondly, for the joint estimator FGL, Danaher *et al.* have provided an explicit and computationally efficient solution. Simulation studies shows that PLNFGL performs better than traditional GGMs and other models designed for scRNA-seq data. In real-data application, PLNFGL effectively processes scRNA-seq data and uncovers meaningful gene interactions. ESEA brings new insights into pathogenesis of AD and NSCLC by identifying key interaction pathways and potential disease-associated targets.

Despite these strengths, we acknowledge several limitations that warrant future methodological development. First, the conditional dependencies inferred by PLNFGL are undirected and cannot establish causality. Incorporating causal inference methods, directed graphical models, or external perturbation data represents an important direction to further enhance biological interpretability. Second, as applied to spatial transcriptomics, the current PLNFGL model does not account for spatial correlation. Consequently, correlated expression among nearby cells may be confounded by shared microenvironmental signals. The graphs derived from spatial data should therefore be interpreted with caution, and extending the framework to incorporate spatial and temporal coordinates remains a crucial next step. Third, certain components of the framework, particularly the semi-positive definite projection and the fused graphical lasso, may face computational challenges when applied to large gene sets or to datasets involving more than five conditions. Finally, we emphasize that the gene-gene interactions inferred by PLNFGL serve primarily as data-driven hypotheses, which ultimately require further validation through biological experiments.

## Supplementary Material

btag485_Supplementary_Data

## Data Availability

The scRNA-seq data for Alzheimer’s disease is available from the Gene Expression Omnibus (GEO) under accession number GSE138852. The non-small cell lung cancer spatial transcriptomics data can be accessed from the NanoString website (http://nanostring.com/CosMx-dataset). The PLNFGL R package is publicly available at https://github.com/jijiadong/PLNFGL, with an archival version deposited on Zenodo (Zhai and Ji 2026).
