# Supplementary material for: PLNFGL: joint estimation of multi-condition gene networks from single-cell RNA-seq data
Source: Bioinformatics. 2026 Jul 3;42(7):btag485. doi: 10.1093/bioinformatics/btag485 (PMC13384064; doi:10.1093/bioinformatics/btag485)
Supplement: btag485_Supplementary_Data [file btag485_supplementary_data.zip › Supplementary Data.pdf]

Supplementary Data

S1 Edge set enrichment analysis using curated biological interactions

In the edge set enrichment analysis, pathway information was incorporated to evaluate whether the inferred gene-gene interactions were overrepresented within biologically coherent functional units. In the primary edge set enrichment analysis presented in the main text, KEGG pathways were systematically converted into fully connected undirected graphs. This methodological choice was intentionally adopted to facilitate the discovery of novel gene-gene interactions, ensuring that undocumented but statistically significant regulatory relationships were not prematurely excluded from the analysis. To verify that the substantial densification of the background graph resulting from this approach did not artifactually influence our biological findings, we performed a secondary validation analysis utilizing a strictly curated, biologically annotated interaction network as the background edge set.

To construct this biologically grounded background, we replaced the fully connected pathway construction with curated pairwise interactions extracted from public pathway databases (Han *et al.* 2015). This process was restricted to interactions with clear structural or biological support (e.g., physical interactions, regulatory relationships, modifications, and bindings). The resulting background network contains 8,894 genes and 164,826 curated interactions.

We repeated the edge set enrichment analysis for both the Alzheimer's disease (AD) and non-small cell lung cancer (NSCLC) datasets using this curated interaction-based background network, the results of which are presented in **Fig. S1** and **Fig. S2**, respectively. Our results revealed that the significantly enriched pathways identified through this approach were a subset of those detected using the fully-connected background. This indicates that employing a fully-connected background does not compromise the identification of interactions with established biological evidence, while concurrently enabling the discovery of novel enriched pathways.

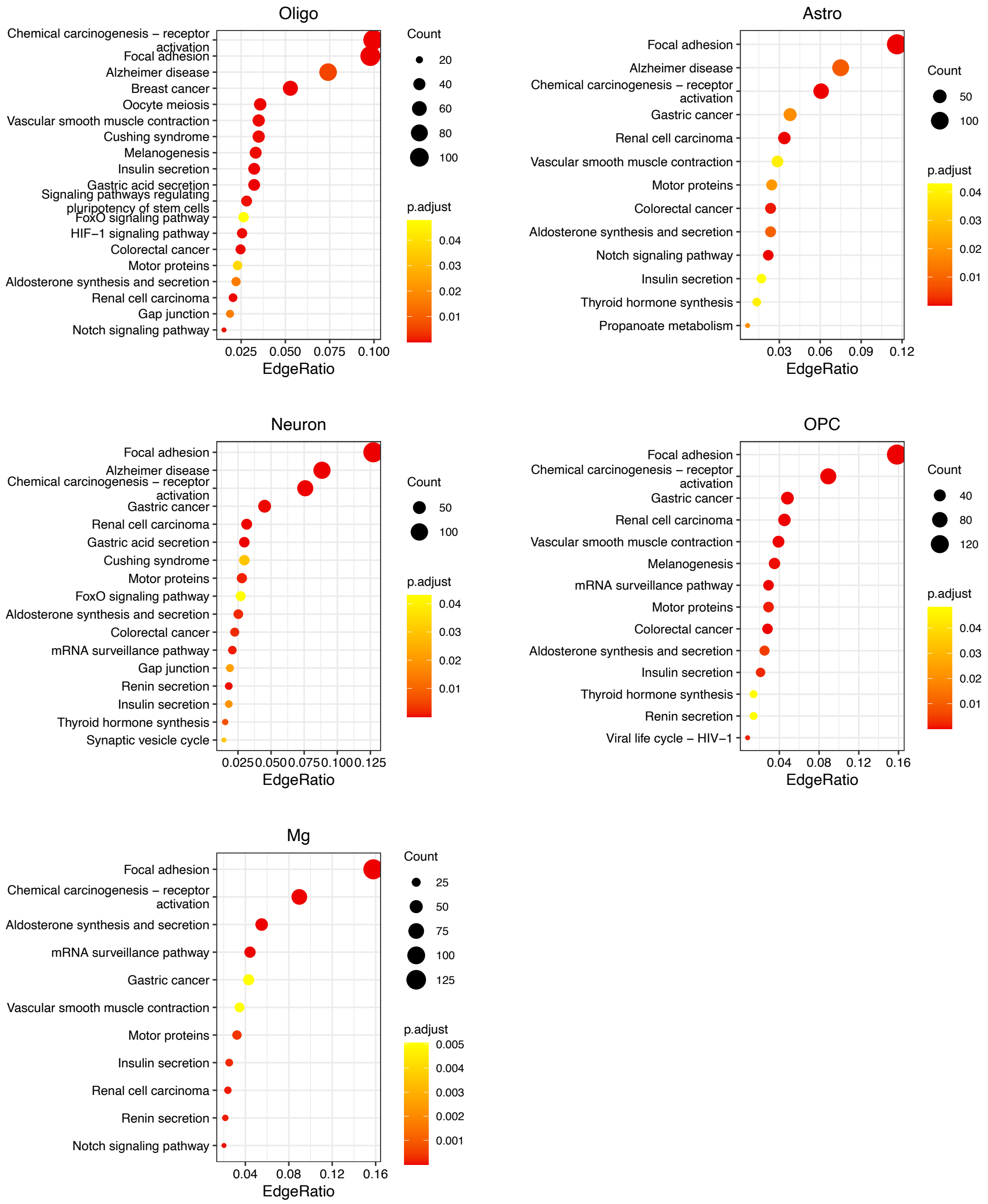

Fig. S1. Significantly enriched pathways identified by ESEA for each cell type, based on biologically supported edges.

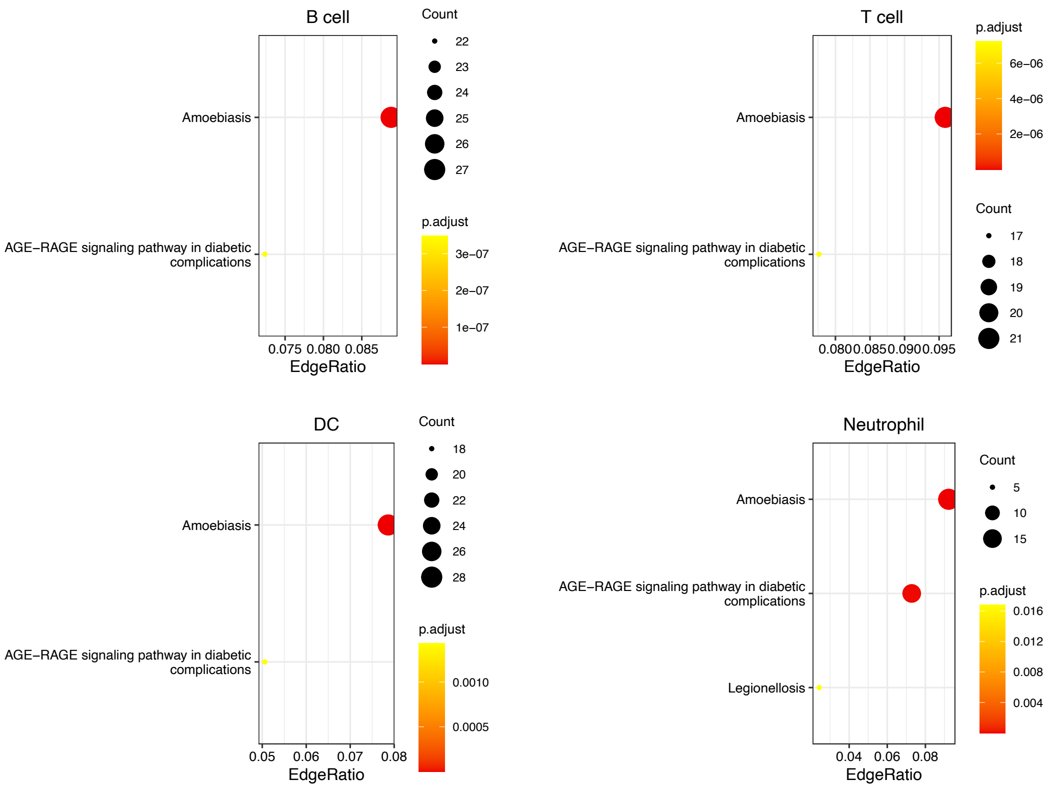

Fig. S2. Significantly enriched pathways identified by ESEA for each cell type, based on biologically supported edges.

S2 Performance for different variations of library sizes

Library size, also known as sequencing depth, is an important consideration in the design of scRNA-seq experiments. In genomic studies of complex diseases, gene expression variations lead to a high degree of heterogeneity in transcriptome-wide depth, making library size an important determinant of estimation accuracy. To assess the impact of library size variation, we conduct additional simulations by adjusting the variance of the distribution that $S_i^{(k)}$ follows, i.e., $\log\left(S_i^{(k)}\right) \sim N\left(\log 10, \sigma^2\right)$, with σ = 0.1 or 0.3 respectively represent low and high variations of library sizes across samples.

As shown in **Fig. S3**, the three network estimation methods designed for scRNA-seq data, namely PLNFGL, PLNet, and JGNsc, demonstrate greater robustness to library size variation compared to GGM-based methods. The library size variation has almost no effect on the former, while the three methods belonging to the Gaussian hypothesis have significantly poorer estimation performance when the library size variation become higher, regardless of whether they estimate separately or jointly. These findings underscore the importance of developing specialized probabilistic models for scRNA-seq data, as such models substantially improve the identification and accuracy of gene regulatory network structures in the presence of sequencing depth variability.

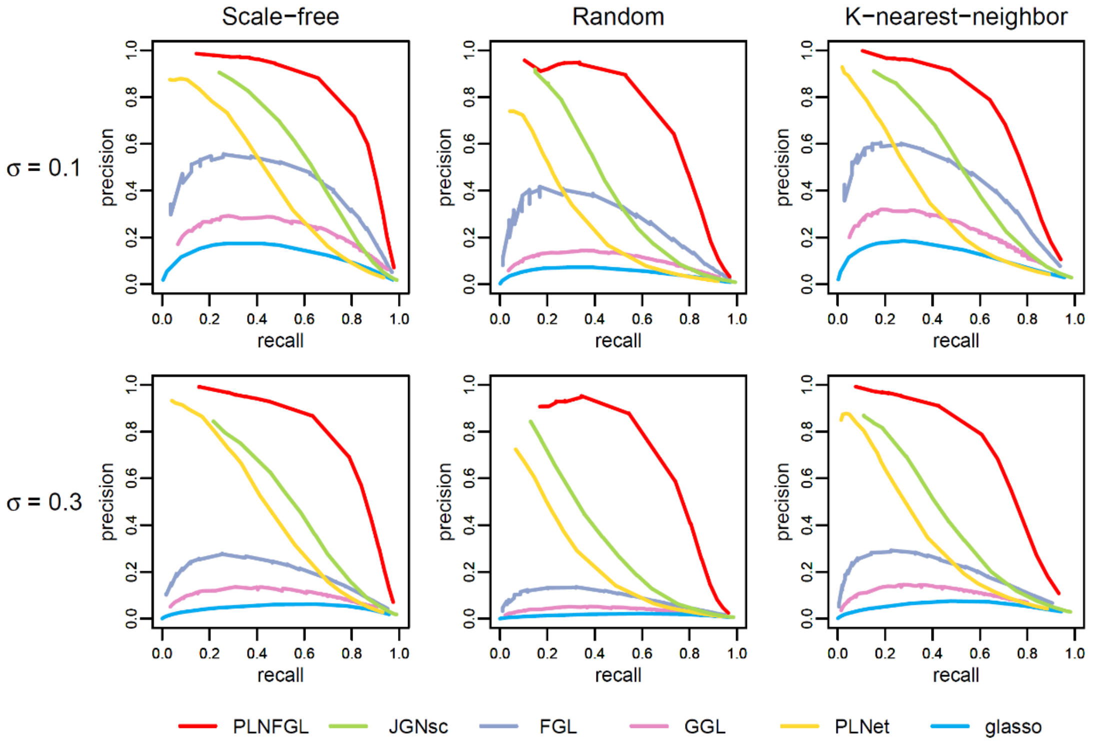

Fig. S3. **Simulation performance of different methods under varying variations of library sizes.** Simulation settings were fixed at n = 200, p = 100, K = 3, dropout rate of 0.3, with σ = 0.1, 0.3. Rows correspond to different variations of library sizes (i.e., σ), while columns represent different underlying graph structures.

S3 Sensitivity analysis for the edge selection threshold

To evaluate the robustness of our results to the choice of the threshold for determining non-zero edges, we performed a supplementary simulation study. In the primary analysis, an estimated edge $\hat{\theta}_{ij}^{(k)}$ was treated as non-zero if $\left|\hat{\theta}_{ij}^{(k)}\right| > 10^{-3}$. Here, we considered three additional threshold values 10^{-4}, 5×10^{-4}, and 5×10^{-3}, while holding all other simulation parameters constant.

The results of the sensitivity analysis are shown in **Fig. S4**. Across all scenarios, the estimated precision and recall for each method varied only minimally across the three thresholds. Crucially, the relative performance rankings among the compared methods remained completely consistent across all threshold values. The comparative advantages demonstrated by PLNFGL were preserved under every condition. These findings confirm that Our simulation results hold for a reasonable range of cutoffs.

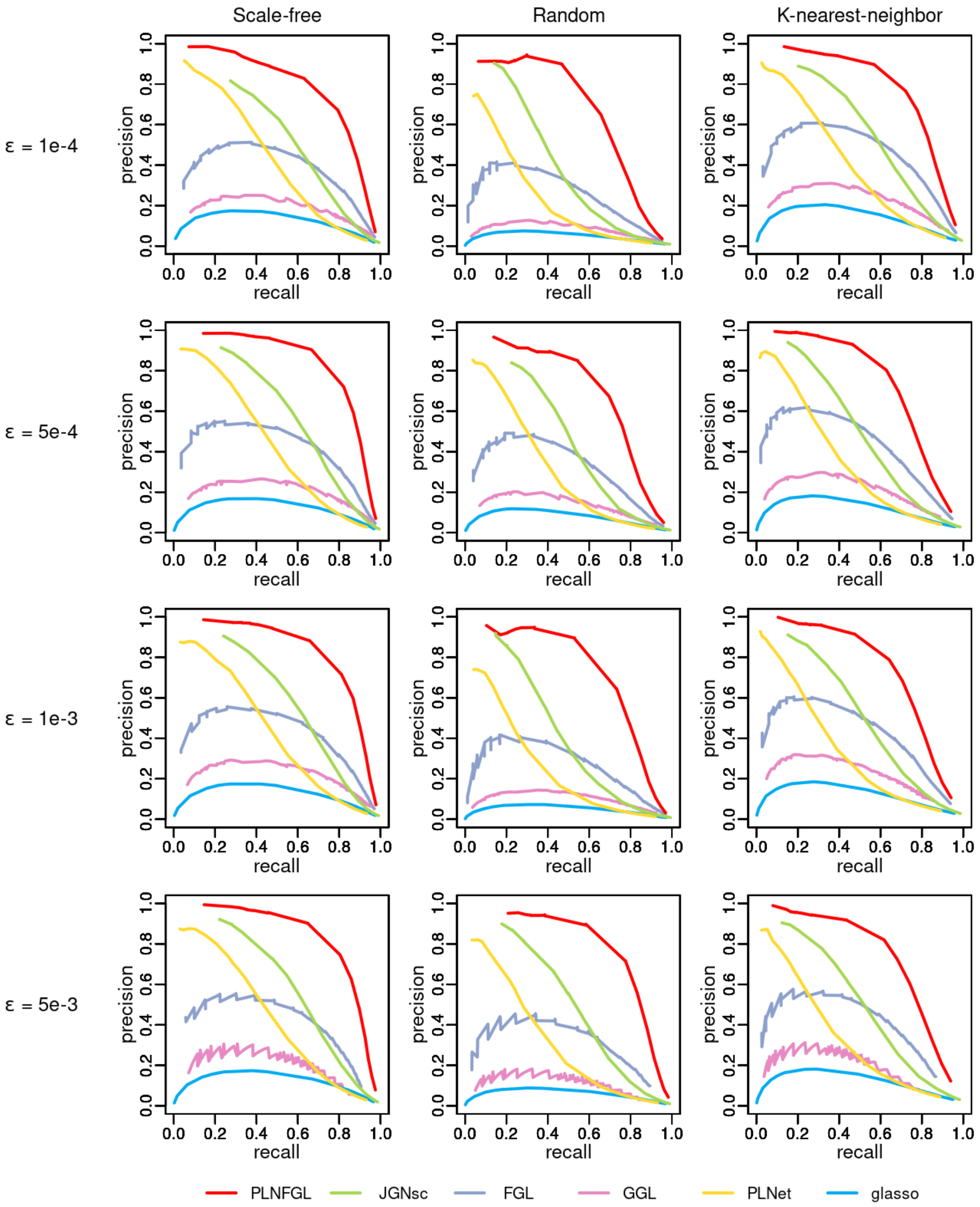

Fig. S4. Simulation performance of different methods under varying edge selection threshold values. Simulation settings were fixed at $n = 200$, $p = 100$, $K = 3$, dropout rate of 0.3, with edge selection threshold (i.e., ε.) of 10^{-4}, 5×10^{-4}, 10^{-3}, and 5×10^{-3}. Rows correspond to different edge selection thresholds, while columns represent different underlying graph structures.
